# Rickettsioses as Underrecognized Cause of Hospitalization for Febrile Illness, Uganda

**DOI:** 10.3201/eid3109.250479

**Published:** 2025-09

**Authors:** Paul W. Blair, Sultanah Alharthi, Andrés F. Londoño, Abdullah Wailagala, Yukari C. Manabe, J. Stephen Dumler, the Acute Febrile Illness and Sepsis in Uganda Study Teams

**Affiliations:** Vanderbilt University Medical Center, Nashville, Tennessee, USA (P.W. Blair, S. Alharthi); Uniformed Services University, Bethesda, Maryland, USA (A.F. Londoño, J.S. Dumler); Makerere University, Kampala, Uganda (A. Wailagala); Johns Hopkins University School of Medicine, Baltimore, Maryland, USA (Y.C. Manabe)

**Keywords:** Rickettsial infections, rickettsioses, bacteria, vector-borne infections, bacterial infections, sepsis, epidemiology, tick-borne, molecular diagnostic techniques, spotted fever group, typhus group rickettsia, sub-Saharan Africa, Uganda

## Abstract

The complexity of rickettsial serodiagnostics during acute illness has limited clinical characterization in Africa. We used archived samples from sepsis (n = 259) and acute febrile illness (n = 70) cohorts in Uganda to identify spotted fever and typhus group rickettsiae by using immunofluorescence assay and clinically validated rRNA reverse transcription PCR (RT-PCR). Among 329 participants, 10.0% had rickettsial infections (n = 33; n = 20 identified with immunofluorescence assay and n = 13 by RT-PCR). Serum rRNA RT-PCR was 75.0% (95% CI 42.8–94.5%) sensitive and 91.2% (95% CI 85.8–95.1%) specific. Thrombocytopenia was more common among patients with rickettsial infections than with other nonmalarial infections (adjusted odds ratio 3.7; p = 0.003). No participants were on a tetracycline antimicrobial drug at admission. rRNA RT-PCR is a promising diagnostic strategy for identifying acute rickettsial infections. Doxycycline should be included in empiric antimicrobial drug regimens for nonmalarial febrile illness in this region.

Rickettsial infections are challenging to clinically distinguish from other causes of febrile illness. Clinical, operational, and technical factors increase the difficulty of identifying rickettsioses in sub-Saharan Africa (*1*). Rickettsial infections in sub-Saharan Africa are of international importance; among returning travelers from the region, rickettsioses are common causes of nonmalarial fever (*2*–*5*). Many patients do not have an obvious eschar (*5*–*7*), and clinical signs and symptoms are not well-characterized among those hospitalized in the region (*5*,*8*). Characteristic laboratory abnormalities of thrombocytopenia, leukopenia, and elevated transaminase activities are commonly observed clinical manifestations of the most prevalent tropical infections, including malaria, typhoid fever, arboviruses, or generalized sepsis (*9*).

Clinicians do not have reliable diagnostics for acute rickettsioses (*10*,*11*). Empiric antimicrobial drug regimens rarely include antimicrobial drugs active against rickettsioses (e.g., tetracyclines) (*11*–*13*). Diagnostics are generally limited to acute- and convalescent-phase serology despite flaws in performance, sparse point-of-care availability, and almost absent acute care clinical utility (*10*). Because of the low organism concentration within the bloodstream in acute infection, serum or whole blood PCR is generally insensitive at 18% (*10*). rRNA, on the other hand, is highly abundant, conserved, and stable. Targeting rRNA with reverse transcription PCR (RT-PCR) is an approach that has been used to optimize analytical sensitivity in PCRs for rickettsial (*14*) and other low bacterial burden infections (*15*). Evaluations using archived samples from patients with rickettsioses and nonrickettsial diseases demonstrate real promise for improved detection sensitivity of rickettsial infections but have yet to be evaluated in samples from prospectively identified acute febrile illnesses in a comprehensive clinical study (*14*,*16*). To confirm the improved sensitivity of this approach, we developed and evaluated new primers targeting rRNA. We describe the performance of an rRNA-targeting RT-PCR to detect spotted fever group (SFG) rickettsiae and typhus group (TG) rickettsiae, compared with acute- and convalescent-phase immunofluorescence assays (IFA), among acute febrile hospitalized participants in 3 hospitals in rural Uganda. We used the results from those identified rickettsial infections to describe the features of hospitalized rickettsial infections and address a clinical epidemiology knowledge gap in this region.

## Materials and Methods

According to internal review board–approved parent cohort protocols, participants were enrolled upon hospitalization. We collected demographic, symptom, examination finding, and laboratory data on standardized forms during hospitalization and at 1 month after enrollment (Appendix) (*13*,*17*; P.W. Blair et al., unpub. data, https://www.medrxiv.org/content/10.1101/2023.09.14.23295526v1). We collected acute blood samples at enrollment and convalescent samples at 1 month. We determined survival during in-person visits or telephone calls during a 1–3-month period after hospitalization in the acute febrile illness (AFI) cohort and a 12-month period in the sepsis cohort.

We used acute-phase serology from archived samples collected at parent study enrollment to determine seroprevalence in the sepsis (n = 311) and AFI (n = 122) cohorts. We conducted IgG IFA by using commercial slides (spotted fever group rickettsia, *Rickettsia conorii* Malish 7 strain; typhus group rickettsia, *Rickettsia typhi* Wilmington strain) (Biocell Diagnostics Inc.). We screened serum samples collected at time of hospitalization (acute phase) and at 1-month follow-up (convalescent phase) at a dilution of 1:64 and titrated to 1:65,536 (*17*). We considered a sample seropositive at a threshold titer of >128. We used a titer of 32 for fold-change calculations if the screen was negative. We defined a confirmed case as a seroconversion with a >4-fold increase in titer from the acute to convalescent sample, in which the convalescent titer was >128. If a participant had a seroconversion to both SFG and TG rickettsiae, we determined the group designation by the higher convalescent titer and then the higher acute titer. We excluded rickettsial infections with an alternative nonrickettsial positive microbiological result on the basis of our protocolized testing (malaria antigen positive, tuberculosis PCR positive or urine lipoarabinomannan positive, positive blood cultures, or positive whole blood Biofire panel) from this analysis to decrease the risk for misclassification (Appendix Figure 1).

For RT-PCR, we extracted RNA (targeting mRNA and rRNA to optimize sensitivity) (*10*) from 200 µL acute serum and 200 µL acute whole blood by using QIAamp RNA Mini Kits (QIAGEN). We conducted RT-PCR by using previously published methods (*18*) targeting SFG rickettsia *sca0* and TG rickettsia 17-kDa outer membrane lipoprotein mRNA. We only considered samples positive if in duplicate. To develop the 16S rRNA primers and probes, we aligned genes across 41 rickettsial species and 17 clinically relevant nonrickettsial bacterial species downloaded from GenBank by using the MEGAX platform (*19*). We identified a conserved region that was disparate from nonrickettsial species, including the forward primer 5′-gcgggtaatgccgggaactataag-3′, reverse primer 5′-ccgaactgagatgtcttttaggg-3′, and probe 5′-/56-FAM/gccggagga/zen/aggtggggacgacgtc/3IABkFQ/-3′. To determine primer species specificity or exclusivity compared with off-target organisms, we compared detection and SYBR Green melting curves by using 14 rickettsial and 4 nonrickettsial DNA controls. We used a 175-bp target sequence cloned into the pCR2.1 vector as a plasmid for quantification with a quantitative PCR master mix (Bio-Rad Laboratories). We identified a cutoff for rRNA RT-PCR by using the lowest threshold with >90% detection among serially diluted whole blood samples and in serum samples from healthy donors spiked with cell-free *R. parkeri*. To determine RT-PCR clinical sensitivity and specificity, we used IFA seroconversions as the index comparator. We prioritized samples with limited volume for IFA and then 16S RT-PCR, *sca0*, and 17-kDa RT-PCR. The rRNA RT-PCR performance calculations were limited to those with sample availability for both complete paired IFA and RT-PCR testing for serum and whole blood (n = 172 participants).

### Case–Control Analysis

To evaluate for differences in clinical parameters, groups included samples with a follow-up serologic test and we then divided them into groups of rickettsial infections (IFA seroconversion or acute RT-PCR positive [*sca0*, 17-kDa, or whole blood 16S rRNA]), malarial infections (on the basis of a rapid diagnostic test), and nonmalarial infections (no rickettsial seroconversion and a negative malaria test). We performed summary statistics for baseline characteristics and microbiology results. We used Kruskal-Wallis testing for continuous parameters, Fisher exact test for observations <5, and χ^2^ test for categorical parameters. We assessed groups for balance of age, female sex, and a >2 quick sequential organ failure assessment (qSOFA) score by using Fisher exact test. We compared rash and clinical laboratory parameters (white blood cell count, platelet count, aspartate transaminase, alanine transaminase, and creatinine) between the rickettsial group and the malarial group and between the rickettsial group and the nonmalarial group. The sample size was insufficient for clinical comparisons of total participants with discordant PCR and IFA serology results. Because of performance limitations of the index IFA comparator, we also determined the performance when limiting negative cases to those with microbiologically confirmed nonrickettsial infections as a secondary analysis.

To determine the discrimination accuracy for identifying rickettsial infection from nonmalarial illness, we conducted multivariable logistic regression among covariates with a significant (p<0.05) result from analyses for rickettsial compared with nonmalarial illness (ultimately limited to platelet count) adjusted for age, sex, and parent cohort. We obtained receiver operating characteristic curve estimates by using 3,000-fold cross-validation. We did not perform a multiple comparisons correction because of sample size. We estimated an effect size of an odds ratio of 2.6 to be detectable with a statistical power of 80% with a 1-sided α <0.05 comparing 33 rickettsial infections with 8:1 matching. We conducted analyses by using Stata version 16.0 (StataCorp, LLC), and created figures by using Stata or R version 4.0.1 (The R Project for Statistical Computing).

## Results

### Serology

**Figure 1:**
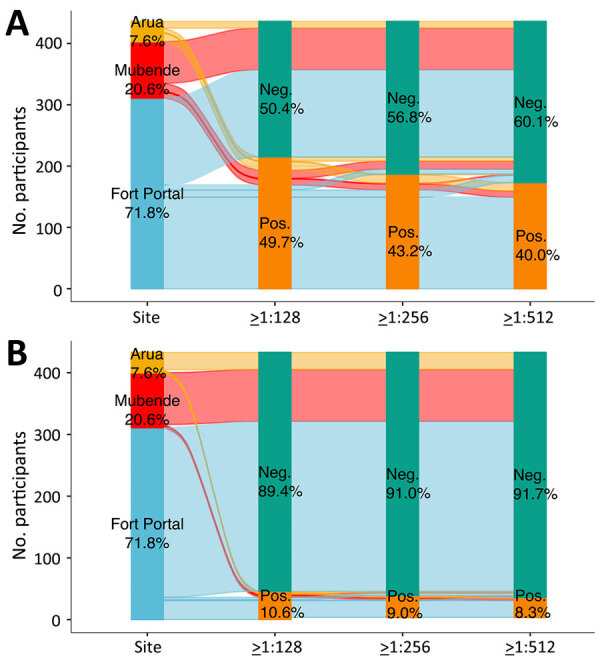
Alluvial plots of baseline acute serum samples from study of rickettsioses as an underrecognized cause of hospitalization for febrile illness, Uganda. A) Spotted fever group rickettsiae; B) typhus group rickettsiae. Immunofluorescence assay IgG seroprevalence is shown for different sites and different titer cutoffs. Participant samples were from referral hospital clinical study sites in Arua (in yellow; 7.6% of participants), Mubende (in red, 20.6% of participants), and in Fort Portal (in blue, 71.8%). Distribution of the colored lines across the graph shows a comparison of positive or negative samples among the sites. Green is the total percentage of negative samples. Orange is the total percentage of positive samples. Neg, negative; pos, positive.

In the SFG, the median time from acute-phase sample collection to convalescent-phase sample collection was 28 days (interquartile range [IQR] 24–30 days). We found that 49.7% (215/433) of acute samples and 58.5% (196/335) of convalescent samples were seropositive (>128) for SFG rickettsia. Among acute samples, 43.2% (187/433) were positive at >256 and 40.0% (173/433) were positive at >512 (Figure 1, panel A). Among samples with a positive screen (titer >64), the median acute titer was 1,024 (up to 131,072; IQR 128–4,096) and median convalescent titer was 2,048 (up to 131,072; IQR 512–8,192). Baseline acute-phase sample seropositivity was highest in the city of Arua (acute 66.7% [22/33]), followed by Fort Portal (acute 54.3% [169/311]) and Mubende (acute 27.0% [24/89]). Among samples with a positive screen, the acute geometric mean titer (GMT) was 1,137.6 (95% CI 877.0–1,475.5) and the convalescent GMT was 1,990.5 (95% CI 1,508.1–2,627.2).

In the TG, we found that 10.6% (46/433) of acute-phase samples and 13.4% (45/335) of convalescent-phase samples were seropositive (>128) for TG rickettsia. Among acute samples, 9.0% (39/433) were positive at >256 and 8.3% (36/433) were positive at >512 (Figure 1, panel B). Among samples with a positive screen, the median acute titer was 512 (up to 65,536; IQR 128–4,096) and median convalescent titer was 2,048 (up to 131,072; IQR 512–8,192). Compared with SFG rickettsia serology, baseline acute phase sample TG seropositivity varied less across sites, with the highest prevalence of seropositive results in Arua (acute: 15.2% [5/33]), followed by Fort Portal (acute 11.6% [36/311]), and Mubende (acute: 5.6% [5/89]). Among positive screens, the acute GMT was 873.4 (95% CI 486.3–1,568.6) and convalescent GMT was 1,915.9 (95% CI 1,030.6–3,561.8). After excluding samples with multiple positive nonrickettsial and rickettsial results (n = 14) (Appendix Figure 1), we observed SFG seroconversions (>4-fold rise in titers) among 4.4% (14 participants) and TG seroconversions among 1.9% (6 participants) of participants.

### rRNA RT-PCR Analytical Validation

To determine primer species specificity compared with off-target organisms, we conducted SYBR Green melting curves that were positive among 8 SFG species and *R. typhi* archived DNA controls and negative for 4 nonrickettsial DNA controls, 2 *Anaplasma* spp., and 2 *Ehrlichia* spp. (Appendix Table 1). We identified a16S rRNA RT-PCR cycle threshold (Ct) cutoff of 35.0 as the lowest threshold to detect >90% of serially diluted *R. parkeri* spiked samples in both whole blood and serum. This threshold equated to a lower limit of detection of 61 genomic equivalents/mL in whole blood and 3.8 genomic equivalents/mL in serum (Appendix Table 2). Healthy control (n = 6) samples were negative.

### rRNA RT-PCR Compared with IFA Seroconversion Cases

Among participants with complete IFA testing and sufficient volume, acute-phase whole blood and serum were available for nucleic acid extraction and RT-PCR from 172 participants (including 12 with seroconversion). Compared with IFA seroconversion as the index test, rRNA RT-PCR was 33.3% sensitive (95% CI 33.3–65.1%; 4 of 12 cases) by using whole blood and 75.0% sensitive (95% CI 42.8– 94.5%; 9 of 12 cases) by using serum. Among rickettsial rRNA RT-PCR positive seroconversion positive cases, serum Ct values were 30.2–34.4 for serum and 29.8–31.9 for whole blood.

### Discrepant Cases 

Among cases without IFA seroconversion, 4/160 whole blood samples were rRNA RT-PCR positive (97.5% specific [95% CI 93.7%–99.3%]; Ct range 31.7–34.8) and 14/160 serum samples were rRNA RT-PCR positive (91.2% specific [95% CI 85.8%–95.1%]; Ct range 29.9–34.5) (Appendix Figure 2). Half or more (7 of 14 serum and 3 of 4 of whole blood) of the discrepant cases had an acute titer higher than the 95% CI of the GMT mean for the cohort. The time between the acute-phase and convalescent-phase sample collection was 23–49 days among positive serum cases and 23–29 days among positive whole blood cases. If restricting the nonrickettsial case definition to microbiologically confirmed diagnoses (n = 62), whole blood 16S rRNA was 100% specific (95% CI 94.2%–100.0%; 0 positive nonrickettsial cases) and serum 16S rRNA was 96.8% specific (95% CI 88.8%–99.6%). Two cases without >4-fold change in IFA under this definition were rRNA RT-PCR positive and were infected with malaria. One had acute and convalescent SFG IgG titers of 65,636 (seroconversion was not observable because of the titration upper limit).

### *Rickettsia* mRNA RT-PCR Compared with IFA Seroconversion Cases

Among participants with both acute and convalescent serum samples, 1 was *Rickettsia* mRNA RT-PCR (17-kDa) positive. This case was also rRNA positive. On the basis of this single case, *sca0* and 17-kD protein gene transcript RT-PCR targets combined (positive with either target) were 14.3% sensitive (95% CI 0.4%–57.9%; 1 of 7 IFA cases) and 100.0% specific (95% CI 97.7%–100.0%). All samples were negative for 17-kDa or *sca0* targets when using PCR on whole blood with or without reverse transcription or on serum without reverse transcription.

### Clinical Case–Control Rickettsial Comparisons

Participants used in the case-control comparison (n = 329) were a median of 39.0 (IQR 27–54) years of age at enrollment and 62% were female and 38% male (Table 1). Empiric tetracycline antimicrobial drugs were started across cohorts among 5.8% participants. Infection groups had similar distributions of age (p = 0.39), sex (p = 0.72) and a positive qSOFA score (p = 0.77).

**Table 1:** Baseline demographics for case–control comparison in study of rickettsioses as an underrecognized cause of hospitalization for febrile illness, Uganda*

Characteristic	Sepsis cohort, n = 259	AFI cohort, n = 70	Total, n = 329
Age, y, median (IQR)	30.5 (24.0–47.0)	30.5 (24.0–47.0)	39.0 (27.0–54.0)
Sex, no. (%)			
F	160 (62)	43 (61)	203 (62)
M	99 (38)	27 (39)	126 (38)
HIV-positive, no. (%)	95 (37)	23 (33)	118 (36)
Physiologic parameters, median (IQR)			
Heart rate, beats/min	101.0 (90.0–112.0)	109.0 (93.0–116.0)	101.0 (90.0–114.0)
Temperature, °C	37.5 (36.9–38.1)	38.1 (38.0–38.8)	37.6 (36.9–38.3)
Breaths/min	28.0 (24.0–32.0)	20.0 (18.0–24.0)	26.0 (22.0–32.0)
Oxygen saturation	95.0 (92.0–97.0)	98.0 (97.0–99.0)	95.0 (93.0–98.0)
qSOFA >2, no. (%)	53 (21)	12 (17)	65 (20)
Tetracycline treatment, no. (%)	18 (6.9)	1 (1.4)	19 (5.8)
Rickettsial† treatment, no. (%)	126 (49)	18 (26)	144 (44)

*AFI, acute febrile illness; IQR, interquartile range; qSOFA, quick sepsis organ failure assessment. †Antimicrobial drugs with potential rickettsial activity: tetracyclines, macrolides, or quinolones.

On the basis of the high specificity of whole blood rRNA, serum *sca0*, and serum 17-kDa RT-PCR, we expanded our serologic case definition for a case–control comparison to include those assays (Appendix Figure 1). Among patients with acute-phase samples with or without convalescent serum samples, 8.0% (33/412; 20 patients identified with a >4-fold change in IFA) had rickettsial infections. To evaluate for differences in clinical parameters, we made comparisons among groups of rickettsial infections, malarial infections on the basis of a rapid diagnostic test (n = 59), and nonmalarial infections (no rickettsial seroconversion and a negative malaria test, n = 237).

Among those with confirmed rickettsial infections, severity was similar to nonrickettsial infections; 22% had a qSOFA of >2 (Table 2). No participants with rickettsial infections were on tetracycline treatments, and a minority (36%) had received a potentially active antimicrobial drug (i.e., macrolide, quinolone, or chloramphenicol) against rickettsial infections. Rash (including maculopapular because of *R. conorii* infection) (*7*) was observed in only 6% of participants.

**Table 2:** Clinical characteristics among those with rickettsial, malarial, and nonmalarial illness in study of rickettsioses as an underrecognized cause of hospitalization for febrile illness, Uganda*

Characteristic	Rickettsial, n = 33	Malarial, n = 59	p value, rickettsial vs. malarial†	Nonmalarial, n = 237	p value, rickettsial vs. nonmalarial†
Age, y, median (IQR)	37.0 (28.0–47.0)	35.0 (24.0–51.0)	0.794	40.0 (28.0–55.0)	0.393
Sex					
F	21/33 (64)	39/59 (66)	0.812	143/237 (60)	0.716
M	12 (36)	20 (34)	0.812	94 (40)	0.716
HIV-positive	10/33 (30)	17/59 (29)	0.880	91/237 (38)	0.368
Physiologic parameters, median (IQR)					
Heart rate, beats/min	102.0 (91.0–110.0)	102.0 (92.0–113.0)	0.782	101.0 (89.0–114.0)	0.696
Temperature °C	38.0 (37.1–38.8)	38.0 (37.0–38.8)	0.964	37.5 (36.9–38.1)	0.083
Breaths/min	24.0 (20.0–29.0)	24.0 (20.0–32.0)	0.954	28.0 (24.0–32.0)	0.057
Oxygen saturation	95.0 (93.0–98.0)	97.0 (94.0–98.0)	0.134	95.0 (92.0–97.0)	0.535
qSOFA ≥2, no. (%)‡	7/33 (21)	13/59 (22)	0.927	45/236 (19)	0.770
Tetracycline treatment	0/33 (0)	0/59 (0)	>0.999	19/237 (8.0)	0.142
Rickettsial treatment	12/33 (36)	14/59 (24)	0.197	118/237 (50)	0.148

*Values are no. positive/no. tested (%) except as indicated. IQR, interquartile range; qSOFA, quick sepsis organ failure assessment. †Fisher exact test, Wilcoxon rank-sum test, or Pearson χ^2^ test. ‡Missing for 1 of 328 participants.

**Figure 2:**
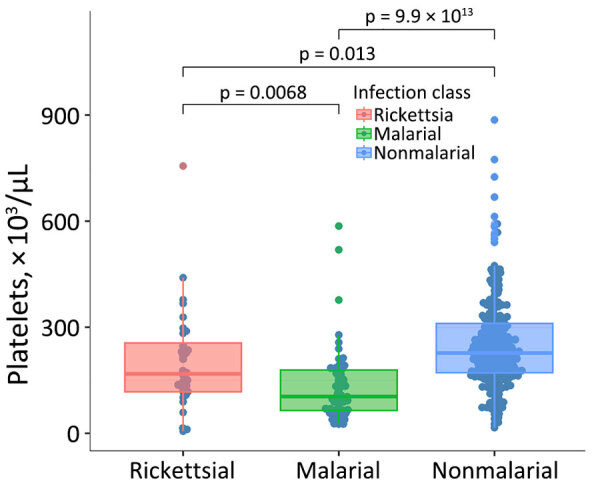
Box plot of platelet counts from patient samples by infection type in study of rickettsioses as an underrecognized cause of hospitalization for febrile illness, Uganda. Horizontal lines within boxes indicate median; box tops and bottoms indicate interquartile ranges; error bar above is 1.5x the IQR to the highest value and the error bar below is 1.5x the IQR to the lowest value.

Among patients with rickettsial illness, median leukocyte count was 6.2 × 10^3^ cells/µL (IQR 4.6–8.1 × 10^3^ cells/µL), aspartate transaminase 38 U/L (IQR 26.0–92.0 U/L), and platelet count 168.0 × 10^3^/µL (IQR 116.9–264.5 × 10^3^/µL) (Table 3). Among SFG seroconversions, the median acute titer was 384 (IQR 128–2,048) and median convalescent titer was 1,536 (IQR 1,024–16,384). Among TG cases, the median acute titer was 64 (IQR 32–8,192) and median convalescent titer was 4,096 (IQR 1,024–65,536). Thrombocytopenia was more common among those with rickettsial infections compared with other nonmalarial infections (adjusted odds ratio 3.7; p = 0.003), but diagnostic performance was limited (sensitivity 45.5%, 95% CI 24.4–67.8%; specificity 83.6%, 95% CI 78.1–88.1%; positive likelihood ratio 2.8, 95% CI 1.6–4.8; negative likelihood ratio 0.7, 95% CI 0.4–1.0). The cross-validated area under the receiver operating characteristic curve was 0.68 (95% CI 0.46–0.74). In addition, the platelet count was lower among patients with malarial illness than rickettsial illness (p = 0.007) (Table 3; Figure 2). Leukocyte count was higher among patients with rickettsial infections than malarial illness (median 4.7 × 10^3^ cells/µL, IQR 3.3–6.6 × 10^3^ cells/µL; p = 0.013). Other parameters were not significantly different. Of participants with rickettsial infections, 3 of 33 died within 90 days.

**Table 3:** Clinical examination and laboratory features among diagnostic classes in a study of rickettsioses as an underrecognized cause of hospitalization for febrile illness, Uganda *

Characteristic	Total, n = 329	Malarial, n = 59	Rickettsial, n = 33	p value, rickettsial vs. malarial†	Nonmalarial, n = 237	p value, rickettsial vs. nonmalarial†
Rash on examination, no. (%)‡	21/328 (6.4)	2/59 (3.4)	2/33 (6.1)	0.616	17/236 (7.2)	>0.999
Leukocytes, × 10^3^ cells/µL§	5.5 (3.9–8.4)	4.7 (3.3–6.6)	6.2 (4.6–8.1)	0.012	5.6 (4.1–9.4)	0.900
Platelet count, × 10^3^ cells/µL ¶	205.0 (134.7–289.0)	104.1 (64.2–179.0)	168.0 (116.9–264.5)	0.007	227.0 (171.0–311.0)	0.013
AST, U/L ‡#	35.0 (26.0–59.0)	32.0 (24.0–42.0)	38.0 (26.0–92.0)	0.081	36.0 (26.0–61.0)	0.524
ALT, U/L	23.0 (18.0–36.0)	21.0 (17.0–28.0)	25.0 (19.0–44.0)	0.119	24.0 (18.0–37.0)	0.596
Creatinine, mg/dL**	0.8 (0.5–1.0)	0.8 (0.0–1.0)	0.8 (0.0–1.1)	0.204	0.8 (0.6–1.0)	0.541

*Values are median (interquartile range) except as indicated. ALT, alanine transaminase; AST, aspartate transaminase; qSOFA, quick sepsis organ failure assessment. †Fisher exact test, Wilcoxon rank-sum test, or Pearson χ^2^ test. ‡No. positive/no. tested (%). Missing for 1 of 329 participants. §Missing for 18 of 328 participants. ¶Missing for 19 of 328 participants. #Missing for 1 of 329 participants. **Missing for 2 of 322 participants.

## Discussion

Our study found that rickettsioses were a common cause of hospitalized illness across multiple sites in Uganda, consistent with a high observed seroprevalence. Rickettsial illness manifested similarly to nonmalarial illness on the basis of well-described clinical parameters. Clinical factors (including platelet count) were neither sensitive nor specific for identifying rickettsial infections. Rashes were infrequently reported or seen on physical examination, and no eschars were observed. Specific rickettsial treatments were uncommonly used. rRNA RT-PCR sensitivity in serum samples was considerably higher than targeting rickettsial mRNAs, although the use of rRNA RT-PCR has the potential benefit as a diagnostic strategy for identifying acute cases that otherwise would not be treated. Our findings highlight the need for continued diagnostic development. Clinicians should also have a high level of suspicion and low threshold for empiric doxycycline use in this region for nonmalarial illness among hospitalized adults.

Among prior cohorts in Sub-Saharan Africa, clinical descriptions of rickettsial infections have largely been limited to returning travelers, and the course of hospitalized illness is not prospectively well characterized. The high rates of seroprevalence observed and common identification of seroconversions indicate that rickettsial infections are circulating widely in this region. A high number of rickettsial infections is supported by a recent study that identified SFG rickettsia in ticks throughout Uganda (*20*). We found rickettsial infections were common (8%) among patients with acute febrile illness or sepsis manifestations. This result is similar to an estimate from 2012–2014 among pediatric and adult febrile participants in Tanzania in which 8.9% had SFG rickettsia seroconversions (*21*). Our findings greatly strengthen the evidence that rickettsial infections frequently cause hospitalization in this region.

Novel acute diagnostics are needed to identify rickettsioses. Diagnostic delays for rickettsial infections often result in prolonged hospital stays and increased death because rickettsial infections are not susceptible to standard empiric antimicrobial drugs used in many worldwide settings (*2*,*22*,*23*). The current diagnostic standard, indirect IFA, relies on referral acute and convalescent serum sample confirmation testing, which is only available 3–6 weeks after symptom onset and has performance limitations (*9*). Our observations of rRNA RT-PCR–positive IFA-negative participants highlighted challenges with using a 4-fold IFA titer increase. In addition to logistical barriers, IFA interpretation could be affected by blood collection timing (*24*), early receipt of antimicrobial drugs (*25*), and immunocompromising conditions (*26*). Characteristics of rickettsial infection were not reliable as clues for initiating empiric treatment, further demonstrating the diagnostic gap and need to consider empiric treatment without fully relying on clinical laboratory results in the absence of a sensitive rapid diagnostic test.

We demonstrated potential improvement in sensitivity by using RT-PCR rather than PCR and by using highly abundant and stable rickettsial rRNA targets rather than membrane protein gene DNA or mRNA targets. Molecular amplification typically has low sensitivity (≈7%–43%) for rickettsioses because of low organism burden; sensitivity can be improved by extracting nucleic acid from the buffy coat, which requires additional processing (*10*,*18*). By using multiple singleplex RT-PCR primers (targeting membrane protein gene DNA or mRNA) we were able to detect cases not identified with DNA PCR. However, sensitivity remains limited with either RT-PCR or PCR by using traditional targets present in either low numbers or that are labile. We anticipated whole blood would contain more rickettsial organisms and improve sensitivity compared with serum across PCR targets. Instead, we found that whole blood was less sensitive than serum, potentially because of inhibitors in whole blood (*27*). rRNA RT-PCR improved sensitivity of PCR over membrane protein gene DNA and mRNA targets and demonstrated promise as an acute detection diagnostic modality that detected rickettsial cases missed by DNA, but mRNA detection was not higher than anticipated when compared with previous studies using DNA (*18*).

Our results are supported by prior efforts that showed improved performance by using 23S rRNA targets (*14*,*16*). The stability and abundance of protein-associated rickettsial rRNA targets are promising for use in remote settings where sample handling and storage can be more vulnerable to unstable conditions. However, rRNA still lacks sensitivity compared with paired serologic testing. Scalable methods to improve specificity and potentially sensitivity such as digital droplet PCR or concentrating target templates in each reaction are needed before broad clinical use (*18*). However, because RT-PCR is now widely available, potentially with increased capacity after the COVID-19 pandemic, and doxycycline is generally well tolerated, laboratory-validated rRNA RT-PCR could be used to initiate standard of care treatment with doxycycline and then serologic confirmation. Rickettsial rRNA genes are abundant stable targets that could be leveraged for furthering clinical rickettsial diagnostics, but implementation and validation studies are needed at clinical sites. Although performance could be optimized, rickettsial rRNA RT-PCR might be the best option for clinical use in highly endemic regions in the absence of a reliable acute diagnostic alternative.

The first limitation of our study is that it relied on availability of convalescent-phase serologic testing to identify approximately half of the infected patients. Therefore, the severity of each group is not anticipated to be representative of the population because participants lost to follow-up or who had died before 28 days were not included. Second, in emphasizing sample availability for multimodal case identification, our study was not designed to determine differences in deaths or duration of hospital stay. Third, sample collection, handling and storing conditions at resource-limited clinical sites might have decreased PCR sensitivity. rRNA RT-PCR performance could be improved with direct testing after collection, although rRNA is likely less vulnerable to degradation than mRNA. Last, conserved rRNA targets afford broad detection of rickettsioses but are intrinsically less phylogenetically or taxonomically informative, prohibiting the possibility for species level identification of SFG rickettsiae.

In conclusion, we found rickettsial infections to be common across 2 severe infectious illness cohorts in Uganda. Our case–control study identified that commonly suggested clinical factors for identifying rickettsial infections in sub-Saharan Africa are nonspecific or are generally absent. rRNA RT-PCR improved sensitivity over previously used membrane protein gene DNA PCR and mRNA RT-PCR and requires further clinical validation to ensure specificity when using conserved stable rRNA targets. Until acute diagnostics are widely available for rickettsial infections, empiric doxycycline should be considered for nonmalarial fever of unknown cause in this region.

Appendix Additional information about rickettsioses as underrecognized cause of hospitalization for febrile illness, Uganda.
