# Supplementary material for: Rickettsioses as Underrecognized Cause of Hospitalization for Febrile Illness, Uganda
Source: Emerg Infect Dis. 2025 Sep;31(9):1708–17. doi: 10.3201/eid3109.250479 (PMC12407214; doi:10.3201/eid3109.250479)
Supplement: Appendix — Additional information about rickettsioses as underrecognized cause of hospitalization for febrile illness, Uganda. [file 25-0479-Techapp-s1.pdf]

Article DOI: https://doi.org/10.3201/eid3109.250479

EID cannot ensure accessibility for supplementary materials supplied by authors. Readers who have difficulty accessing supplementary content should contact the authors for assistance.

Rickettsioses as Underrecognized Cause of Hospitalization for Febrile Illness, Uganda

Appendix

Supplementary Methods

After presentation to the emergency department or outpatient department and designation for hospitalization, patients received informed consent in IRB-approved parent protocols. Rash was assessed as part of a clinical exam consistent with standard practice (excluding a full skin exam) and prospectively recorded if present using a standardized report form. Clinical tests were routinely performed including complete blood counts and chemistries. Microbiologic testing included blood culture with antimicrobial sensitivity testing, HIV testing, malaria smears and rapid diagnostic tests; all were routinely performed as previously described (*1*) (P.W. Blair, et al., unpub. data, https://www.medrxiv.org/content/10.1101/2023.09.14.23295526v1). If there was clinical suspicion, PCR testing for tuberculosis was performed using expectorated sputum (Cepheid) and participants living with HIV had a urine lipoarabinomannan (LAM test). Whole blood was run on the FilmArray Global Fever (BioFire) panel for 19 non-rickettsial pathogen targets (*2*).

Appendix Table. Exclusivity testing with SYBR-green PCR using DNA from multiple clinically relevant species. Isolates with a PCR curve and melting curve separate from the non-template control indicated as positive.

Group	Species	Result
Spotted fever group rickettsia	*R. akari*	Positive
	R. conorii	Positive
	R. felis	Positive
	R. montanensis	Positive
	R. parkeri	Positive
	R. peacockii	Positive
	R. rickettsii	Positive
	R. siberica	Positive
Typhus group rickettsia	*R. typhi*	Positive
Ehrlichia spp.	*E. chaffeensis*	Negative
	E. muris eauclairensis	Negative
Anaplasma spp.	*A. capra*	Negative
	A. phagocytophilum	Negative
Orientia spp.	*O. tsutsugamushi*	Negative
Candida spp.	*Pichia kudriavzevii* (formerly *Candida krusei*)	Negative

Group	Species	Result
Enteric bacteria	*E. coli*	Negative
	Ps. aeruginosa	Negative
Staphylococcus spp.	*S. aureus*	Negative

References

1. Blair PW, Kobba K, Kakooza F, Robinson ML, Candia E, Mayito J, et al. Aetiology of hospitalized fever and risk of death at Arua and Mubende tertiary care hospitals in Uganda from August 2019 to August 2020. BMC Infect Dis. 2022;22:869. PubMed

2. Manabe YC, Betz J, Jackson O, Asoala V, Bazan I, Blair PW, et al. Clinical evaluation of the BioFire Global Fever Panel for the identification of malaria, leptospirosis, chikungunya, and dengue from whole blood: a prospective, multicentre, cross-sectional diagnostic accuracy study. Lancet Infect Dis. 2022;22:1356–64. PubMed
